# *Helicobacter pylori*-Positive Gastric Biopsies—Association with Clinical Predictors

**DOI:** 10.3390/life12111789

**Published:** 2022-11-04

**Authors:** Anca Negovan, Andreea-Raluca Szőke, Simona Mocan, Claudia Bănescu

**Affiliations:** 1 Department of Clinical Science-Internal Medicine, “George Emil Palade” University of Medicine, Pharmacy, Science, and Technology of Târgu Mureș, 540139 Târgu Mureș, Romania; 2 Department of Pathophysiology, “George Emil Palade” University of Medicine, Pharmacy, Science, and Technology of Târgu Mureș, 540139 Târgu Mureș, Romania; 3 Pathology Department, Mureș County Clinical Hospital, 540011 Târgu Mureș, Romania; 4 Pathology Department, Emergency County Hospital of Targu Mures, 540136 Târgu Mureș, Romania; 5 Genetics Laboratory, Center for Advanced Medical and Pharmaceutical Research, “George Emil Palade” University of Medicine, Pharmacy, Science and Technology of Târgu Mureș, 540139 Târgu Mureș, Romania

**Keywords:** *Helicobacter pylori*, immunohistochemistry, premalignant gastric lesions, proton-pump inhibitors

## Abstract

Introduction: Although *Helicobacter pylori’s* role in gastric oncogenesis is well-known, only a fraction of infected patients develop cancer. Hence, more factors are supposed to be involved. The objectives of the present study were to investigate the impact of clinicopathological parameters on *Helicobacter pylori* status. Methods: The study included 1522 patients referred for endoscopy: study group consisted of 557 patients with *Helicobacter pylori*-positive biopsies confirmed using histochemical stains or immunohistochemistry methods; and the control group consisted of 965 patients with *Helicobacter pylori*-negative status on histology. Results: Severe endoscopic lesions were more frequent in the *Helicobacter pylori* group (*p* < 0.001), with no difference noticed in the distribution of premalignant gastric lesions (*p* = 0.82). Anemia and dyslipidemia were independent factors associated with *Helicobacter pylori*-positive biopsies (*p* < 0.05). Non-steroidal anti-inflammatory therapy was more frequently administered in the study group, while proton-pump inhibitors had an anti-*Helicobacter pylori* activity on histology (*p* < 0.0001). Conclusion: In the studied population, patients with *Helicobacter pylori*-positive biopsies had a more frequent history of gastrotoxic medication, severe endoscopic lesions, and anemia. *Helicobacter pylori* was unpredictable by gastrointestinal symptoms. The frequency of premalignant gastric lesions was similar irrespective of the actual status of infection, underlining the importance of unintentional clearance of bacteria in old infection and the remaining risk for cancer in this population.

## 1. Introduction

Gastric cancer (GC), the fourth leading cause of cancer-related death worldwide, is associated with *Helicobacter (H.) pylori* infection, considered the most consistent etiological factor in both the precursor lesions (atrophic gastritis (AG), intestinal metaplasia (IM)) and subsequent GC development [1,2].

Because of the high prevalence of *H. pylori* and the low survival rate for GC estimated at around 15.5% in eastern Europe, prevention and management of infected patients are still challenges for clinicians [3]. Although *H. pylori* is a recognized cause of GC, only a fraction of infected individuals develop cancer. Hence, in positive *H. pylori* patients, other potential risk factors for GC are supposed to be involved, with the cellular and molecular events and the tumor microenvironment being the mechanisms widely studied in the last decade [4,5,6].

Diagnostic testing for *H. pylori* infection can be divided into non-invasive (serology, stool antigen, and urea breath tests) and invasive methods (culture, histology, and urease tests) when there is an indication for endoscopy [7].

Histology can document *H. pylori* infection and appreciate the degree of inflammation or associated pathology such as AG, IM, dysplasia, or cancer. Special stains like Giemsa and specific immunohistochemical (IHC) methods are used for histopathologic detection of *H. pylori* infection in gastric biopsies [8,9].

The objectives of the present study were to investigate the impact of clinicopathological factors, including gastrointestinal symptoms, chronic gastroprotective and gastrotoxic drug consumption, social habits, comorbidities, mucosal lesions, and premalignant gastric lesions (AG and IM), in patients with positive *H. pylori* status on biopsies (histological/IHC detected) in comparison with patients with negative biopsies.

## 2. Materials and Methods

### 2.1. Study Population

We performed a case-control, retrospective, observational study that enrolled all sequential patients who underwent an esophagogastroduodenoscopy (EGD) between 2018–2021 at Medical Clinic No. 2 in Târgu Mureș Emergency County Hospital, Romania.

All the patients included in the study presented indications for endoscopy because of dyspeptic symptoms (particularly unresponsive to therapy or associated with alarm signs), duodenogastric reflux symptoms, anemia, or endoscopic surveillance for malignity. A subset of patients with cardiovascular diseases underwent an endoscopy to assess the gastrointestinal bleeding risk before initiating the antithrombotic therapy.

Of the 1726 consecutive patients, 204 (11.8%) patients met one of the following exclusion criteria: previous *H. pylori* eradication therapy, severe medical conditions (malignity, active digestive hemorrhages requiring endoscopic hemostasis, severe heart diseases, end-stage renal disease, dementia), and missing data and were excluded from further analysis. The cases with predominant corpus atrophy and positive serology for autoimmune gastritis were also excluded from the present study.

**Scheme 1:**
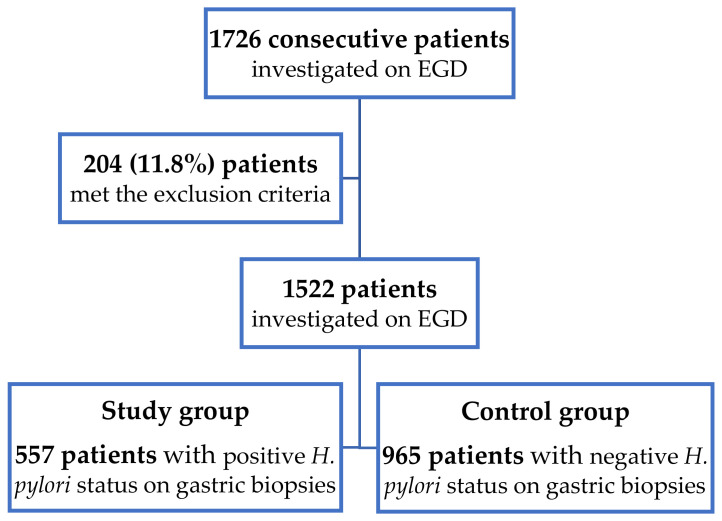
Patients’ selection.

The remaining 1522 patients were divided into two groups: study group—557 patients confirmed with positive *H. pylori* status on biopsy using histochemical stains or immunohistochemistry methods; and control group—965 patients with negative *H. pylori* status on all biopsy sites (Scheme 1).

### 2.2. Data Collection

Medical records and endoscopic and histologic results were reviewed and noted in a designed database.

The current symptoms: epigastric pain, heartburn, regurgitation, flatulence, and nausea/vomiting were registered. Proton-pump inhibitors (PPI) therapy was considered if administered for at least one month before investigation. Non-steroidal anti-inflammatory drugs (NSAIDs—ibuprofen, ketoprofen, dexketoprofen, etc.), antiplatelet drugs (clopidogrel, low-dose aspirin (LDA)), and anticoagulants (antivitamin K, low-weight molecular heparin, and new oral anticoagulants—apixaban, rivaroxaban, dabigatran) were noted if consumed at least one month in regular doses before the upper digestive endoscopy. Data about drug consumption was obtained using both direct interviews and medical records.

The medical history of the patients was checked for comorbidities: respiratory disease (asthma/chronic obstructive pulmonary disease), cardiovascular diseases (ischemic heart disease, congestive heart failure, valvular heart disease), chronic osteoarticular diseases, diabetes mellitus, and cerebrovascular disease (ischemic and hemorrhagic stroke). They were noted as present/absent. Cut-off values of hemoglobin levels according to WHO statements for anemia were considered <12 g/dL in women and <13 g/dL in men, without distinction between the macrocytic and microcytic forms [10]. Dyslipidemia was defined as elevated total (≥240 mg/dL) or low-density lipoprotein (≥190 g/dL) cholesterol levels associated or not with hypertriglyceridemia (>150 mg/dL) [11].

Social behavior (alcohol, tobacco smoking) was considered if patients declared consumption of at least ten units (10 mL) of pure alcohol weekly and >5 cigarettes/day, including smoking cessation in the past five years.

### 2.3. Endoscopic and Histologic Findings

During the endoscopic examination, the presence of reflux esophagitis, hiatal hernia, and biliary reflux were noted. The Los Angeles Classification System was used [12] in the endoscopic assessment of reflux esophagitis. Patients with endoscopic appearance of esophageal columnar metaplasia lined at least 1 cm in a circular or tongue shape (suspected as Barrett’s esophagus) were also included. Reflux esophagitis was recorded in the study as present or absent.

We used a modified Lanza Score for the evaluation of endoscopic findings [13]. Grade 0: no visible gastro-duodenal lesions; Grade 1: one erosion or petechiae (hemorrhagic area without mucosal defect); Grade 2: 2 to 10 erosions/petechiae; Grade 3: more than 10 erosions/petechiae; and Grade 4: more than 25 erosions/petechiae or stomach ulcer. Patients with Lanza scores of 3 and 4 were registered with severe endoscopic lesions.

Five gastric tissue biopsies were obtained according to the protocol of Sydney for *H. pylori* detection: two from the antrum, two from the corpus (the greater and the lesser curvature), and one from the angular notch and sent in 5 different containers. Fixed formalin biopsies were embedded in paraffin, and 3-5 microns sections were processed and stained with Hematoxylin and Eosin (H&E), PAS-alcian blue, and modified Giemsa. Chronic inflammation of the gastric mucosa, activity, AG, and IM were assessed according to the Updated Sydney System. AG and complete and incomplete IM were considered premalignant gastric lesions.

The diagnosis of reactive gastropathy relied on histologic features such as foveolar hyperplasia, mucin depletion in surface epithelial cells, fibromuscular replacement of the lamina propria, and capillary congestion.

Patients were considered to have *H. pylori*-negative status when all the biopsy specimens were negative. *H. pylori*-positive status was defined if the bacteria were identified in at least one gastric tissue. IHC was performed when *H. pylori* was not seen with H&E or Giemsa stains, but there were histological features that raised the suspicion of *H. pylori* infection: significant active gastritis and patients with PPI therapy associated with persistent inflammation. The immunohistochemistry technique for *H. pylori* detection used FLEX polyclonal rabbit anti-*H. pylori* antibody, Ready to Use (DakoAutostainer), and EnVision FLEX, High pH as a visualization system.

### 2.4. Statistical Analysis

Continuous variables are presented as the median and interquartile range (IQR). Categorical data are summarized using frequencies and percentages. Fisher’s exact test was utilized for the comparison of categorical data; the Mann–Whitney U-test was applied for the comparison of continuous variables. All significant factors and factors whose unadjusted estimated significance level was *p* ≤ 0.25 in univariate analysis were selected as candidates for multivariate logistic regression. The partial likelihood ratio test was applied to decide the analyzed variables in the final model. All tests were two-sided. A *p*-value < 0.05 was considered statistically significant. All data were computed using the JASP (JASP graphical program for statistical analysis supported by the University of Amsterdam, Amsterdam, The Netherlands) program v.0.12.1.

## 3. Results

The prevalence of *H. pylori*-positive biopsies among the study participants was 57.7%, the majority of them (95%) having pangastritis on histology. From 243 (15.9%) cases for which IHC was performed, 49 biopsies (20.1%) were positive for *H. pylori*. Patients diagnosed with positive *H. pylori* status were statistically significantly younger: median (Q1–Q3) 61 years (51–70) for cases vs. 64 years (53–72) for controls, U = 293387, *p* = 0.0029. Homogenous sex distribution in both the study and the control group was observed (Table 1).

All the investigated symptoms (epigastric pain, heartburn, nausea/vomiting, bloating) had a similar frequency between the two groups (all *p* > 0.05). No association was noticed between *H. pylori* and endoscopic findings such as reflux esophagitis, biliary reflux, and hiatal hernia (*p* > 0.05).

Severe mucosal lesions (Lanza scores 3 and 4) were more frequent in patients with positive *H. pylori* biopsies; the relationship was maintained in univariate and multivariate logistic regression analysis (*p* < 0.001) (Table 2).

The histologic assessment of the control group demonstrated 380 (39.4%) patients with reactive gastropathy, 388 (40.2%) patients with chronic inactive gastritis, 66 (6.8%) with chronic active gastritis, and 131 (13.6%) without histologic lesions. There was no difference noticed in terms of premalignant gastric lesions (AG and intestinal IM) between the two groups (*p* > 0.05).

The distribution of other coexisting conditions (cardiovascular, respiratory, chronic kidney diseases, or diabetes mellitus) was similar (all *p* > 0.05). Cardiovascular diseases had the highest prevalence of all studied chronic conditions, with 626 (41.3%) patients from all the study participants.

Patients with histologically positive *H. pylori* status presented more frequent dyslipidemia, which remained an independent factor in multivariate logistic regression analysis (*p* = 0.03) (Table 3). Anemia was associated with *H. pylori* in the multivariate regression model, irrespective of concomitant NSAIDs and severe endoscopic lesions (*p* < 0.01). When included in a logistic regression model alongside other chronic diseases associated with systemic inflammation (diabetes mellitus, chronic kidney disease, cardiovascular diseases, cerebrovascular disease, and chronic respiratory diseases), the relationship between anemia and *H. pylori* remained significant (*p* = 0.03).

NSAID treatment was statistically significantly more common in the *H. pylori* group, while PPI therapy was negatively associated with the bacterial load on general histochemical stains (*p* < 0.001). Compared with their counterparts, patients with *H. pylori* in biopsy samples were recorded more often as smokers and with a positive history of alcohol consumption in univariate but not multivariate regression analysis (*p* > 0.05).

**Figure 1:**
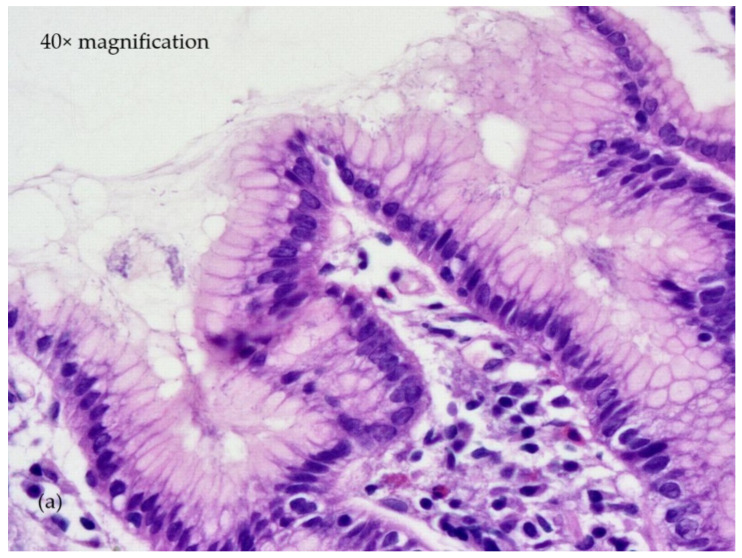

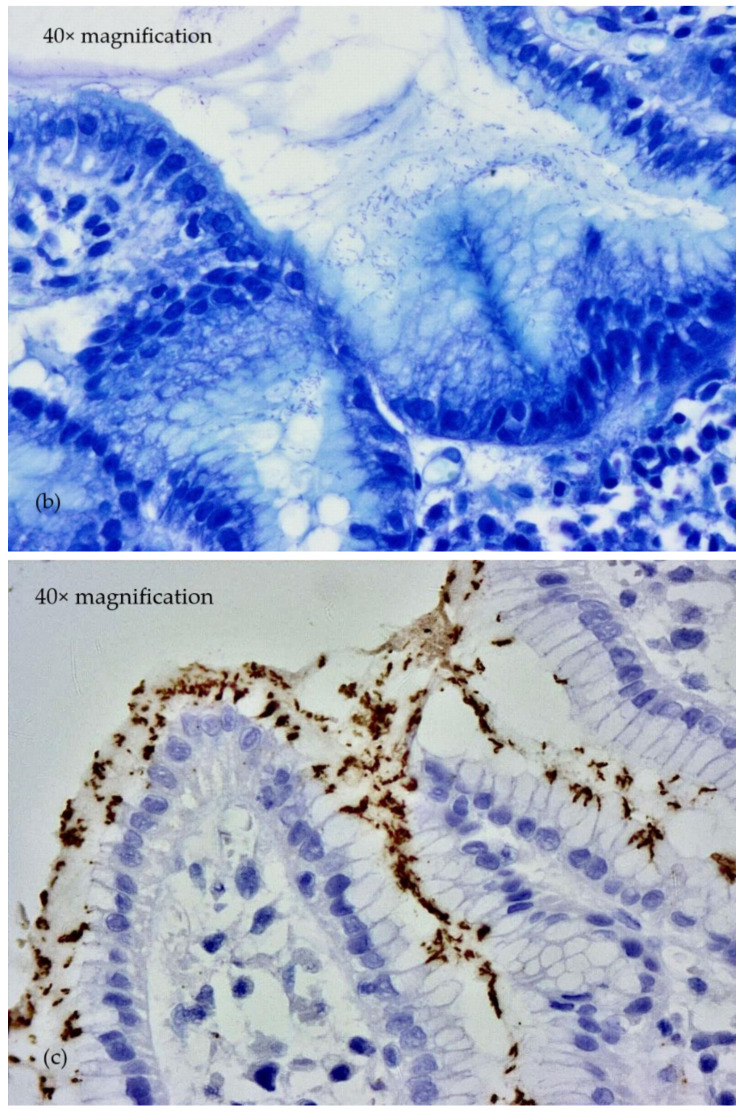
(**a**) Histologic aspects in *Helicobacter pylori* gastritis, Hematoxylin and Eosin staining; magnification, ×40. (**b**) Positive Giemsa stain for *Helicobacter pylori* microorganisms, magnification, ×40. (**c**) Immunohistochemical staining for *Helicobacter pylori* with numerous positive bacteria attached to the luminal surface of the foveolar cells; magnification, ×40.

The histologic aspects of *H. pylori* gastritis are seen in Figure 1.

## 4. Discussion

It is known that geographical differences in the prevalence of *H. pylori* infection and GC exist in all parts of the world [14]. The prevalence of premalignant gastric lesions (AG and IM) is largely unknown and the discrepancy in study results comes from the selection of cases, lack of data in the general population, evaluation based on different biopsy protocols, or using serologic evaluation for AG. 

Although the prevalence of *H. pylori* infection decreased over the last decades due to improvements in sanitary and eradication methods, recent data from a systematic review demonstrated high *H. pylori* rates in Eastern Europe [3]. These data confirm the validity of *H. pylori*-positive biopsies proportion (57.7%) prevalence in the present study. In Table 4, we compared our results with those reported in other biopsy studies worldwide, supporting the mentioned differences. 

The present study enrolled Romanian, consecutive patients investigated by endoscopy and systematically evaluated with biopsy from the antrum and corpus. Of these patients, almost 40% had premalignant gastric lesions (AG/IM). The proportion is high compared with similar Western studies (11%) but comparable with that published in the endoscopic Asian population (57%), suggesting that the risk of GC in unintentionally eradicated subjects is theoretically equal to that of successfully eradicated cases [15,16,17,18,19].

Histochemical stains are used to evaluate *H. pylori* gastritis, while IHC is recommended in cases with persistent inflammation associated with premalignant gastric lesions (AG, M), PPI therapy, and follow-up biopsies after eradication treatment for *H. pylori* if no bacteria were found using general histochemical stains [20].

It is already known that PPIs can lead to false-negative results on biopsy urease, urea breath, and stool antigen tests because the therapy reduces the number of microorganisms that may only be present in the oxyntic mucosa [20,21,22]. Additionally, because PPI therapy may favor the colonization of the gastric mucosa with numerous other microorganisms due to the low-acid environment, *H. pylori* antibodies are recommended for determining the presence of *H. pylori* among different types of bacteria. However, cases with few bacteria can be missed inclusively in immunohistochemical specimens. Furthermore, other *Helicobacter* species can stain positive with antibodies (especially polyclonal) for *H. pylori*, and morphologic differences are difficult to be made on immunohistochemical stains [21].

Studies highlighted that under unfavorable conditions (alkaline pH, aerobiosis) or exposure to PPIs and various antimicrobial agents, *H. pylori* changes from the bacillary to the coccoid morphology. Furthermore, the latest research suggests that coccoid forms contribute to recurrences after *H. pylori* eradication treatment [23,24]. On the other hand, the coccoid forms should be differentiated from other bacterial cocci and fungi from the stomach. For these purposes, the IHC method can be used because it also stains the coccoid forms of the bacteria [21].

Well-developed countries registered a global decrease in *H. pylori* prevalence, including the younger generation [25], while the study results demonstrated that patients with positive biopsies were younger compared with patients from the control group. Because of aging of the mucosa, PPIs, or antibiotics with anti-*H. pylori* activity [20,21,22], elderly patients with these therapies prescribed throughout life may have an unintentional clearance of the bacteria and may be underdiagnosed using the general histochemical stains.

On the other hand, antibiotic resistance to *H. pylori* infection is of great concern, particularly clarithromycin-resistant strains of *H. pylori*, which are identified in more than 20% of infected patients from central and eastern Europe. In this order, biopsies obtained during esophagogastroduodenoscopy can be used in culture or bimolecular tests evaluating the clarithromycin-resistant *H. pylori* strains [26,27].

In terms of gender, the study shows a similar distribution of *H. pylori* between men and women, in line with other studies from western Europe [3].

The results of our study do not demonstrate any symptoms associated with *H. pylori*. Many positive *H. pylori* patients do not develop dyspeptic symptoms, or the symptoms are transitory. However, *H. pylori* can cause symptoms in some patients. Therefore, *H. pylori* gastritis must be excluded before a diagnosis of functional dyspepsia [28,29,30].

Both NSAIDs and *H. pylori* are independent risk factors for ulcer disease [31]. NSAID treatment and severe endoscopic lesions were statistically significantly more frequent in *H. pylori*-positive biopsies, supporting the importance of *H. pylori* eradication in reducing peptic ulcer bleeding and the development of NSAID-induced mucosal lesions.

*H. pylori* can lead to GC through sequential steps of the Correa precancerous cascade (AG, IM, dysplasia) [2,32]. In the present study, positive status was not associated with the concomitant presence of premalignant gastric lesions (AG and IM). Inflammation caused by *H. pylori* infection damages the structure of gastric mucosa, with subsequent development of AG and IM. As a result, *H. pylori* will disappear because the bacteria cannot survive in the intestinal-type mucosa, native or metaplastic. Corpus atrophy with pseudopyloric metaplasia can resemble antral mucosa; thus, it is essential to distinguish antral mucosa from oxyntic mucosa with extensively glandular atrophy and pseudopyloric metaplasia on the microscopic examination [20,21].

Although in several studies *H. pylori* infection is linked to extra-digestive disorders (coronary heart disease, cerebrovascular and respiratory diseases, diabetes mellitus, end-stage kidney disease) [25,33], there were no statistically significant differences between the groups in the distribution of these comorbidities. In contrast, we found positive *H. pylori* specimens significantly and independently associated with dyslipidemia. Recent epidemiological research reports *H. pylori* as a risk factor for dyslipidemia, arteriosclerosis, and cardiovascular diseases. Additionally, the bacteria promotes the activation of platelets and maintains chronic inflammation, leading to arterial hypertension and endothelial dysfunction [34,35,36].

Anemia remained an independent factor associated with positive biopsies in the multivariate regression model when adjusted for other covariates (severe endoscopic lesions and NSAIDs consumption), supporting the evidence indicating an increased likelihood of iron-deficiency anemia in *H. pylori* gastritis because of reduced acid secretion and iron malabsorption [37]. Additionally, anemia of inflammation develops in patients with chronic conditions that cause persistent immune-cells activation, including chronic pulmonary diseases, congestive heart failure, chronic kidney disease, etc. [38]. Anemia associated with systemic inflammation in chronic diseases was excluded as an error source when analyzed in a multivariate logistic regression alongside diabetes mellitus, chronic kidney disease, cardiovascular diseases, cerebrovascular disease, and chronic respiratory disease.

Both smoking and alcohol, studied in relationship with GC, were found as risk factors for GC development. The existing data about *H. pylori* and smoking suggest an increased persistence of the infection and a higher prevalence in smokers [39,40,41]. In this study, the smoking role was suppressed by the other variables when included in the logistic regression model, suggesting that additional factors are necessary to facilitate the *H. pylori* infection. Chronic alcohol abuse is reported to reduce bacteria acquisition [42]. Statistics revealed that alcohol consumption was positively associated with *H. pylori* in the univariate, but not in multivariate regression analysis.

The present study included a subset of patients with an increased frequency of cardiovascular diseases and gastrotoxic medication belonging to a population from eastern Europe with a still high prevalence of *H. pylori*. The results have shown that positive gastric biopsies were not associated with specific digestive symptoms, suggesting the low predictability of symptoms and the utility of the test-and-treat strategy in young patients with uninvestigated dyspepsia and no alarming symptoms in high prevalence *H. pylori* regions [20,28,30].

Research performed in a European population with increased prevalence *of H. pylori* infection and GC, similar to Asian populations, may contribute to understanding and improving strategies for GC prevention, especially in the selection of cases requiring surveillance. Our results highlight the importance of histological and IHC studies in patients with PPI consumption in whom non-invasive tests for *H. pylori* may lead to false-negative results and delay of treatment, further increasing the risk for cancer.

The study has all the limitations of a retrospective, observational, and single-center study but represents a real-life “perspective” of systematic concomitant assessment of clinical and endoscopic parameters associated with the histopathological findings.

## 5. Conclusions

In the studied population, patients diagnosed with *H. pylori* infection from biopsies reported a more frequent history of gastrotoxic medication and developed severe endoscopic lesions and anemia more often. The infection was unpredictable by dyspepsia or other symptoms. In patients without previous eradication therapies for *H. pylori* infection, the frequency of premalignant gastric lesions was similar irrespective of the actual status of infection, underlining the importance of unintentional clearance of bacteria in old infection and the remaining risk for cancer in this population.

## Figures and Tables

**Table 1:** Group differences between demographic and clinical characteristics.

Parameter	Study Group (Positive *H. pylori* Status) N = 557		Control Group (Negative *H. pylori* Status) N = 965		*p*-Value *	OR	95% CI
	n	%	n	%			
Male gender	293	52.6	463	47.9	0.0627	1.22	0.99 to 1.50
Age > 50 years	426	76.4	771	79.8	0.1196	0.81	0.63 to 1.05
Endoscopic and histologic findings							
Reflux esophagitis	119	21.3	204	21.1	0.94	1.01	0.78 to 1.30
Severe endoscopic lesions	130	23.3	156	16.1	<0.001	1.57	1.21 to 2.04
Biliary reflux	195	35.0	342	35.4	0.91	0.98	0.78 to 1.22
Hiatal hernia	164	29.4	312	32.3	0.25	0.87	0.69 to 1.09
Premalignant gastric lesions ^a^	213	38.2	378	39.1	0.82	0.97	0.78 to 1.20
Comorbidities							
Cardiovascular diseases	225	40.3	401	41.5	0.66	0.95	0.77 to 1.17
Cerebrovascular diseases	38	6.8	38	3.9	0.01	1.78	1.12 to 2.83
Chronic respiratory diseases	83	14.9	164	16.9	0.31	0.85	0.64 to 1.14
Diabetes mellitus	105	18.8	159	16.4	0.26	1.17	0.89 to 1.54
Anemia	110	19.7	226	23.4	0.10	0.80	0.62 to 1.04
Dyslipidemia	253	45.4	387	40.1	0.04	1.24	1.00 to 1.53
Drug consumption							
Antiplatelet drugs	213	38.2	326	33.7	0.08	1.21	0.97 to 1.50
Anticoagulants	67	12.0	136	14.0	0.27	0.83	0.60 to 1.14
NSAIDs ^b^	104	18.6	122	12.6	<0.01	1.58	1.19 to 2.11
PPIs ^c^	207	37.1	490	50.7	<0.0001	0.57	0.46 to 0.70
Symptoms							
Epigastric pain	298	53.5	503	52.1	0.63	1.05	0.85 to 1.30
Heartburn	150	26.9	246	25.4	0.54	1.07	0.85 to 1.36
Regurgitation	37	6.6	54	5.5	0.43	1.20	0.77 to 1.84
Nausea/Vomiting	107	19.2	156	16.1	0.03	1.34	1.02 to 1.75
Bloating	130	23.3	206	21.3	0.36	1.12	0.87 to 1.44
Social behaviour							
Tobacco smoking ^d^	105	18.8	130	13.4	<0.01	1.49	1.12 to 1.97
Alcohol consumption ^e^	142	25.4	201	20.8	0.04	1.30	1.01 to 1.66

^a^ Premalignant gastric lesions: atrophic gastritis and/or intestinal metaplasia; ^b^ NSAIDs: non-steroidal anti-inflammatory drugs; ^c^ PPIs: proton-pump inhibitors; ^d^ Over 5 cigarettes/day; ^e^ More than 2 units/day, 1 unit = 10 mL pure alcohol; * Obtained from Chi-square or Fisher’s exact tests; OR: odds ratio; CI: 95% confidence interval; PPI: proton-pump inhibitors.

**Table 2:** Results from univariate binary logistic regression.

Parameter	Statistics Z	*p*-Value *	Crude OR	95% CI
Male gender	1.87	0.06	1.22	0.99 to 1.50
Age > 50 years	−1.56	0.11	0.81	0.63 to 1.05
Antiplatelet drugs	1.75	0.08	1.21	0.97 to 1.50
NSAIDs ^a^	3.16	<0.01	1.58	1.19 to 2.11
PPIs ^b^	−5.11	<0.001	0.57	0.46 to 0.71
Severe endoscopic lesions	3.43	<0.001	1.57	1.21 to 2.04
Cerebrovascular disease	2.45	0.01	1.78	1.12 to 2.83
Dyslipidemia	2.02	0.04	1.24	1.00 to 1.53
Smoking ^c^	2.78	<0.01	1.49	1.12 to 1.97
Alcohol consumption ^d^	2.09	0.03	1.30	1.01 to 1.66
Anemia	−1.66	0.09	0.80	0.62 to 1.04
Hiatal hernia	−1.17	0.24	0.87	0.69 to 1.09

^a^ NSAIDs: non-steroidal anti-inflammatory drugs; ^b^ PPIs: proton-pump inhibitors; ^c^ Over 5 cigarettes/day; ^d^ More than 2 units/day, 1 unit = 10 mL pure alcohol; * Crude *p* values obtained from Wald’s test; Response variable: presence of *Helicobacter pylori* infection; Crude OR: unadjusted odds ratio; CI: 95% confidence interval.

**Table 3:** The multivariable logistic regression model.

Parameter	b ^e^	SE	*p*-Value *	Adjusted OR	95% CI
NSAIDs ^a^	0.47	0.15	<0.01	1.60	1.18 to 2.18
PPIs ^b^	−0.60	0.11	<0.001	0.54	0.43 to 0.68
Severe endoscopic lesions	0.50	0.14	<0.001	1.65	1.24 to 2.19
Dyslipidemia	0.25	0.11	0.03	1.28	1.02 to 1.62
Anemia	−0.39	0.14	<0.01	0.67	0.50 to 0.89
Male gender	0.14	0.11	0.20	1.16	0.92 to 1.46
Smoking ^c^	0.23	0.19	0.21	1.26	0.87 to 1.84
Alcohol consumption ^d^	−0.06	0.17	0.71	0.94	0.67 to 1.31
Cerebrovascular disease	0.49	0.25	0.05	1.64	1.00 to 2.70

^a^ NSAIDs: non-steroidal anti-inflammatory drugs; ^b^ PPIs: proton-pump inhibitors; ^c^ Over 5 cigarettes/day; ^d^ More than 2 units/day, 1 unit = 10 mL pure alcohol; ^e^ Estimated unstandardized regression coefficients; SE: standard error; * Wald’s test adjusted *p*-value; CI: 95% confidence interval.

**Table 4:** Geographic prevalence of *H. pylori* infection and premalignant gastric lesions (AG and IM) in gastric biopsies.

N *	*H. pylori*	AG	IM	Country	Author
1522	57.7%		35.9%	Romania	
368	26.9%	57.3%		China	Zhang et al. [15]
17710			11.7%	US	Huang et al. [16]
4050			13.8%	Turkish	Olmez et al. [17]
585	80.2%	12.6%	15.2%	Iran	Esmaeilzadeh et al. [18]

* N: number of patients included in the study.

## Data Availability

Data can be found with A.-R.S.
